# The coupling coordination between health service supply and regional economy in China: spatio-temporal evolution and convergence

**DOI:** 10.3389/fpubh.2024.1352141

**Published:** 2024-05-07

**Authors:** Jing Deng, Qianwen Song, Huan Liu, Zicheng Jiang, Chengzhi Ge, Dexun Li

**Affiliations:** ^1^School of Hospital Economics and Management, Anhui University of Chinese Medicine, Hefei, China; ^2^Key Laboratory of Philosophy and Social Science of Anhui Province on Data Science and Traditional Chinese Medicine Innovation and Development, Anhui University of Chinese Medicine, Hefei, China; ^3^Office of Chengdu Shuangliu District Maternal and Child Health Hospital, Chengdu, China

**Keywords:** coupling coordination, spatial convergence, kernel density, health service supply, level of economic development

## Abstract

**Background:**

The coordination of health service supply and regional economy is an integral path to promote China’s prosperity.

**Methods:**

Based on the coupling mechanism of health service supply and regional economy, we sampled the data from 30 provinces in China from 2009 to 2021 in this study and constructed the evaluation index system. Additionally, we calculated the coupling coordination degree (HED) of the two through the coupling coordination degree model. We further used the kernel density estimation, Moran’s I index, and spatial β convergence model to assess the dynamic evolution trends, spatial aggregation effect, and spatial convergence characteristics of coupling coordination.

**Conclusion:**

(1) HED in China showed a rising trend during the study period but with large regional differences, forming a gradient distribution pattern of “high in the east and low in the west.” (2) The results of Kernel density estimation show that HED has formed a gradient differentiation phenomenon within each region in China. (3) HED has modeled spatial clustering characteristics during the study period, with high-value clusters mainly appearing in the eastern region and low-value clusters appearing in the northwestern region. (4) There are absolute β-convergence and conditional β-convergence trends in HED in China and the three major regions during the study period, but there is an obvious regional heterogeneity in the control factors. The research provides a reference for accurately implementing policies according to different levels of health service supply and economic development, in addition to narrowing the regional differences of the coupling coordination between the regional economy and health service supply.

## 1 Introduction

Following China’s economic reforms, the country’s economy has witnessed rapid growth. This, however, has led to regional economic disparities due to differences in geographic locations and resource possession, resulting in a pattern where eastern and southern regions economically outpace the western and northern ones (1). Simultaneously, China faces pronounced disparities in health service supply that are attributed to uneven regional economic progression and variances in local health financial subsidies (2). With China’s advancing economy and an aging population, the demand for healthcare services is increasing. Despite this, China’s per-capital healthcare resources still fall behind developed countries like the United Kingdom, the United States, and Japan, presenting a persistent challenge in healthcare service capacity. Notably, the 14th Five-Year Plan (3) highlights the importance of coordinating regional economic development with healthcare service levels for sustainable national growth. It has been found that the regional economy has a positive stimulating effect on improving the level of health service provision (4). First, the level of economic development affects the input of total medical resources, since the larger the regional economic aggregate, the more resources will be invested in medical and health services (5). Second, the layout of economic development also affects the layout of health resources, where the higher the level of economic development, the stronger the attraction of health resources (6). Third, the level of economic development affects the level of medical and health service capacity, where the residents’ demand for health service supply rises alongside economic development, which in turn promotes the active improvement of medical service capacity (7). Health service supply plays the role of guarantor for national economic and social development as its capacity has a positive effect on the population’s health. A healthy population from a better health service supply translates a healthy workforce that improves the nation’s labor productivity, which promotes economic development (8). In other words, healthcare expenditure promotes economic development. The improvement of health service supply capacity needs to be accompanied by government funding and residents’ health consumption expenditure. The funding patterns of which have been shown in studies as able to promote economic growth (9). Therefore, exploring the coordinated relationship and future trends between health service supply and China’s regional economy is vital for country’s development.

Scholars have employed various research methodologies to investigate the bidirectional relationship between the regional economy and health service supply in diverse regions. Research has demonstrated that when health service supply and the regional economy develop in a synergistic, interactive, and synchronized fashion (10), they significantly contribute to the prosperity and development of a region (11).

The coupled coordination degree model effectively unveils interactions between systems and is thus widely utilized in regional economic research (12, 13). Existing literature on the coupling and coordination between health service provision and regional economy primarily explores various regional coupling relationships and their spatial linkages. Notably, scholars have delved into this coupled system relationship from national, regional, provincial, and municipal perspectives. For instance, Liu et al. (14) analyzed this relationship within China’s health and economic systems using the coupled coordination model. Similarly, Zhou et al. (15) investigated the Yangtze River Delta’s health and economic coupling, finding a strong correlation with the region’s economic development. Ma (16) focused on Shandong Province to study this relationship from a provincial viewpoint, while Chi et al. (17) measured the coupled coordination between health and economy at the county level in China.

Furthermore, spatial analysis tools like Moran’s I index, LISA analysis, geo-detector, and fixed-effects models have been employed to gauge the spatial relationship of this coupled coordination. Gong et al. (18) utilized the Moran index and LISA to analyze China’s spatial state of coupled coordination between health and economy. Hui Tang et al. (19) applied geographic probes for a similar study in China, and Li et al. (20) used coupled fixed-effects models to examine the relationship in Central China.

Although these studies have explored the interrelationship between health service provision and regional economy at different spatial scales, focusing mainly on the characteristics of the spatial and temporal distribution of coupling and the presence or absence of spatial correlation, these studies lack further research on the spatial spillover effects of HED. To address this gap, this study examines 30 Chinese provinces using the coupling coordination degree model to analyze the coordination level between China’s health service supply and regional economy. The dynamic characteristics of the coupling’s development are analyzed using the kernel density method. Moreover, Moran’s I index is utilized to verify spatial correlation (21). Finally, this paper employs spatial β-convergence models to explore the spatial spillover effects of influencing factors.

The potential marginal contributions of this study are as follows: (1) It investigates the dynamic evolution and spatial distribution patterns of HED in China and its three major regions, focusing on spatial patterns and agglomeration. (2) The study uses kernel density estimation to elucidate the dynamic development process of HED across different regions. (3) Employing the conditional β-convergence model, it highlights the regional variances in factors influencing HED, providing a robust scientific basis for precise and localized coordination of health service supply and economic development.

## 2 Methods

### 2.1 Indicator selection

This study selected indicators pertinent to health service supply capacity from China’s 14th Five-Year Plan and Healthy China 2030 Strategy (22). Building on existing research and the health industry’s characteristics, four secondary indicators and nine tertiary indicators were chosen. These include human resources for health (23, 24), physical resources for health (25, 26), health service efficiency (27, 28), and disease prevention and control (29, 30), forming an evaluation system to assess China’s health service supply level (31). Health human resources and physical facilities reflect *per capita* healthcare resource supply, indicating a region’s healthcare service capacity. Health service efficiency measures the operational status of health institutions and is a crucial aspect of regional healthcare capacity. Disease prevention and control not only mirror the effectiveness of local healthcare but also depict the impact of economic and social factors on the healthcare environment (32).

The number of medical institutions, the number of beds, and health personnel are the basis of medical resources and can intuitively reflect the supply capacity of medical services. In this study, licensed or assistant doctors, registered nurses, hospitals, and beds were selected as the level-three indicators to measure the supply level of medical and health services based on the number of beds per 1,000 population. Consultation volume and hospitalization volume are the key indicators reflecting the status of healthcare service utilization and influencing factors (33), in this study, the number of outpatient visits to healthcare institutions, the number of patient hospitalizations, and the average number of outpatient visits undertaken by physicians per day were selected as the tertiary indicators to measure the status of healthcare services. The morbidity and mortality rates of infectious diseases reflect the effectiveness of public health services in epidemic prevention in a region (34). In this study, the morbidity and mortality rates of class A and class B infectious diseases were selected as indicators of disease prevention and control.

Furthermore, the regional economy is analyzed not just in terms of size but also the economic structure’s rationality and socio-economic vitality. Drawing on existing literature (35–39) and adhering to principles of scientific rigor, representativeness, and accessibility (35), the study selects eight indicators across economic scale, structure, and socio-economic dimensions for a comprehensive evaluation.

Overall, economic aggregate is an important indicator of the development level of a regional economic scale, which can represent a country or region’s economic strength. In this study, Gross Domestic Product (GDP), local general public budget revenue, fixed asset investment and other indicators are chosen to measure the regional economic aggregate (40). Economic structure is of great significance to the sustainable development of the economy as an adaptable one helps to improve the overall efficiency and competitiveness of the economic system and promotes the healthy development of the economy. This study chooses the proportion of secondary and tertiary industries in GDP and employment to indicate the rationality of regional economic structure (41). Social economy is an indicator that measures the benefits and results of economic activities, which can represent the economic vitality of a country or region and the consumption capacity of residents. This study selects the *per capita* disposable income of the residents and the total consumption of retail goods in the society to represent the social economy of the region (42).

The chosen indicators for assessing health service supply and regional economic evaluation are presented in Table 1.

**Table 1:** Evaluation index system of China’s HED.

First-Grade index	Second-Grade index	Third-Grade index	Unit	Weights	Attribute
Supply level of medical and health services	Human resources for health	Number of licensed or assistant doctors per thousand people	Individuals	0.1	+
		Number of registered nurses per thousand people	Individuals	0.08	+
	Health facility resources	Number of hospitals per thousand people	Unit	0.12	+
		Number of beds in health institutions per thousand people	Unit	0.09	+
	Efficiency of health services	Outpatient visits to medical institutions	Times	0.23	+
		Hospitalization in medical institutions	Times	0.2	+
		Daily visits per doctor	Times	0.14	+
	Disease prevention and control	Mortality rate for statutory category A and B diseases	%	0.02	−
		Maternal mortality rate	%	0.01	−
Level of economic development	Size of economy	GDP	Yuan	0.19	+
		Government budget revenue	Yuan	0.18	+
		Fixed-asset investment	Yuan	0.16	+
	Economic structure	Secondary sector as a share of GDP	%	0.03	+
		Tertiary sector as a share of GDP	%	0.07	+
		Share of employment in secondary and tertiary sectors	%	0.04	+
	Socio- economic	*Per capita* disposable income	Yuan	0.12	+
		Total social retail merchandise consumption	Yuan	0.2	+

### 2.2 Data sources

Data on the numbers of doctors, nurses, beds, hospitals, outpatient visits, hospitalization cases, daily visits per doctor, mortality rate for statutory category A and B diseases, and mortality rate in the level of health service supply subsystem are from the *China Health Statistics Yearbook* (2009–2022) (43). Data on the economy size and socioeconomic data in the level of economic development subsystem are all from the China Statistical Yearbook (2009–2022) (44). The secondary structure sector as a share of GDP and tertiary sector as a share of GDP in Economic structure are from the *China Statistical Yearbook* (2009–2022). The share of employment in secondary and tertiary sectors is from the *China Labor Statistics Yearbook* (2009–2022) (45).

In terms of regional division, this study adopts the criteria of China’s Seventh Five-Year Plan for the division of China’s three major economic zones (Table 2). This criterion is included in the commonly used regional division by the Chinese Bureau of Statistics.

**Table 2:** Distribution of the three major regions in China.

Region	Provinces
Eastern	Beijing, Shandong, Zhejiang, Hainan, Guangdong, Jiangsu, Hebei, Shanghai, Fujian, Liaoning, Tianjin
Central	Hunan, Hubei, Heilongjiang, Jiangxi, Jilin Henan, Shanxi, Anhui.
Western	Yunnan, Inner Mongolia, Qinghai, Sichuan, Ningxia, Guangxi, Chongqing, Shaanxi, Xinjiang, Guizhou, Gansu

### 2.3 Entropy value method

#### 2.3.1 Subsystem score calculation

##### 2.3.1.1 Standardization of indicator data

To mitigate the impact of varying index scales on measurement results, the original data are standardized using the extreme deviation method (46). Equations (1) and (2) are the dimensionless processof positive and negative indicators respectively.


(1) $$\text{Positive}\;\text{indicators}\;:X_{ij}=\frac{V_{ij}-\min_{1\leq i\leq m}\left(V_{ij}\right)}{\max_{1\leq i\leq m}\left(V_{ij}\right)-\min_{1\leq i\leq m}\left(V_{ij}\right)}$$

(2) $$\text{Negative}\;\text{indicators}\;:X_{ij}=\frac{\max_{1\leq i\leq m}\left(V_{ij}\right)-V_{ij}}{\max_{1\leq i\leq m}\left(V_{ij}\right)-\min_{1\leq i\leq m}\left(V_{ij}\right)}$$

Where $X_{ij}$ is the standardized value of the *i*th *j*th indicator, *V_ij_* is the value of the *j*th indicator in item *i* and *m* is one of the indicators studied (47).

##### 2.3.1.2 Calculation of subsystem scores

After normalizing the data, the subsystem score can be calculated (48). Equation (3) will be used to calculate the value of ith subsystem.


(3) $$f_{i}=\sum_{j=1}^{k_{i}}\omega_{i}X_{ij},\left(i=1,2\right)$$

Where (*i* = 1, 2) is the number of indicators in the *i*th subsystem, and *i* = 1 and 2 denote the health care service provision subsystem and the economic development level subsystem, respectively (49).

#### 2.3.2 Coupling coordination degree model

##### 2.3.2.1 Coupling model

The concept of “coupling,” originally derived from physics, refers to the phenomenon of two or more systems or modes of motion influencing each other through interaction to the point of synergy (50). The concept of coupling has also been gradually incorporated into research in the socio-economic field and has been widely applied to research areas such as urban economy/industry, economy/health, and environment/industry (51–56). Regional economy and health service supply are two correlating systems that can influence each other so this paper will utilize the coupling model to measure the interaction between the two.

Drawing on the capacity coupling coefficient model in physics, a coupling degree model consisting of two subsystems of health care service supply and economic development level was established using the following Equation (4):


(4) $$C=\sqrt{\frac{U_{1}\cdot U_{2}}{{\left(U_{1}+U_{2}\right)}^{2}}}$$

Where *C* denotes HED, C∈ [0,1]; $U_{1}$ denotes the health care service provision subsystem; and $U_{2}$ denotes the level of economic development.

##### 2.3.2.2 Coupled coordination degree model

However, the coupling model cannot always reflect the synergistic effect between the level of health service provision and the regional economy. To address this, this study also refers to the Coupled Coordination Degree Model proposed by Liao Chongbin (57) to assess the degree of coupling and coordination between health service supply capacity and regional economic development.

Further building upon the coupling degree, a coupling coordination degree model was set up to measure the degree of coordination of the interactive coupling between the two systems of medical and health service supply and the level of economic development, using the following Equation (5):


(5) $$\left\{ \begin{array}{c} D=\sqrt{C\times T}\\ T=aU_{1}+bU_{\begin{array}{c} 2\\ \; \end{array}} \end{array}\right.$$

Where D denotes HED; T denotes the comprehensive reconciliation index of the health care service supply subsystem and the economic development level, reflecting the overall synergy or contribution between these systems; *a*, *b* is the coefficient to be determined. This study believes that the health care service supply is equally important to the level of economic development and the two should be mutually reinforcing, so take *a* = *b* = 0.5 (58).

Based on the results of related research (59) and the characteristics of the data in this study, D was categorized into seven basic types (see Table 3).

**Table 3:** The types and classes of HED.

Coupling range	Class	Coupling coordination type
[0, 0.3]	1	Severe dissonance
[0.3, 0.4]	2	Mild dissonance
[0.4, 0.5]	3	Impending dissonance
[0.5, 0.6]	4	Barely coordination
[0.6, 0.7]	5	Primary coordination
[0.7, 0.8]	6	Intermediate coordination
[0.8, 1]	7	Good coordination

#### 2.3.3 Kernel density estimation method

As a nonparametric method, kernel density estimation (KDE) has the advantages of strong robustness and weak model dependence. The KDE method can analyze the regional difference characteristics of the observed objects from the characteristics of the distribution pattern, change trend and ductility of the observed objects (60). Therefore, it has been widely used in the study of regional differences when measuring economic development, health service supply, and health level of the population (61–64).

Therefore, in order to better analyze the characteristics of HED development in different regions of China, we used the KDE method (65) to analyze the distribution dynamics and trends of HED with the following Equation (6):


(6) $$f\left(x\right)=\frac{1}{N_{h}}\Sigma_{i=1}^{N}k\left(\frac{x_{i}-x}{h}\right)$$

Where *N* is the number of samples, *h* is the bandwidth, and *x* is the mean.

#### 2.3.4 Spatial autocorrelation

Spatial autocorrelation analysis is often used to determine if there are interdependencies between variables distributed between the same regions. It is also used to measure if there is spatial aggregation between variables (66, 67). This paper employs the global Moran index and local Moran index to assess spatial correlation and agglomeration, respectively (68).

##### 2.3.4.1 Global

The global Moran index can judge the spatial distribution characteristics between variables from the significance level. In which the positive significance sign implies that there is a centralized distribution characteristics between variables, and negative significance characteristics represent the dispersed characteristics. The size of the global Moran index can also determine the size of the spatial association between variables, where the closer the positive value is to 1, the stronger the concentration and the closer the negative value is to −1, the stronger the dispersion (69).


(7) $$I\left(d\right)=\frac{\Sigma_{i=1}^{n}\Sigma_{j=1}^{n}\left(X_{i}-\bar{X}\right)\left(X_{j}-\bar{X}\right)}{S^{2}\Sigma_{i=1}^{n}\sum_{j=1}^{n}W_{ij}}$$

Here, $X_{i}$ is the observed value of region *i* and $X_{j}$ is the observed value of region *j*, $W_{ij}$ is the spatial weight matrix with spatial adjacency of 1. *I*(*d*) > 0 is the spatial positive correlation, which indicates that there is significant spatial clustering of HED.

##### 2.3.4.2 LISA index

To compensate for the fact that global Moran’s I cannot pinpoint the exact spatial location where aggregation or anomalies occur, the local spatial autocorrelation pattern of HED was analyzed using the Local Indicator of Spatial Association (LISA) (70). Local Indicators of Spatial Association (LISA) was used to measure the degree to which the values of spatial unit variables are similar (positive correlation) or different (negative correlation) from the values of neighboring units. Additionally, it can be also used to identify “hot spots” to test the heterogeneity of the data (71) using the following Equation (7):


(8) $$I_{i}=X_{i}^{\prime }\sum_{j=1}^{n}W_{ij}X_{j}^{\prime }$$

Where $X_{i}^{\prime }$ and $X_{j}^{\prime }$ are normalized for observations in regions i and j. Here $I_{i}$> 0 indicates that HED in this region is less different from the neighboring regions. Meanwhile, $I_{i}$< 0 indicates that there is a significant difference in HED in this region.

#### 2.3.5 Spatial beta convergence model

β convergence is based on the growth theory from the neoclassical school of economics, which believes that the HED of backward regions has a faster growth rate and will gradually catch up with the HED of developed regions and that the HHED of each region will converge to a stable, uniform state as time goes by Barro and Martin (72). β-convergence includes absolute convergence and conditional convergence, where conditional β-convergence means that the HED of each region converges to its respective steady-state level after considering the different effects of other initial endowments (73). Additionally, it has been shown that there is a spatial correlation between both regional economy and health service supply (74), so it is necessary to include spatial factors in the consideration of HED and spatial correlation in the convergence test. Since the traditional convergence model has not yet considered the spatial effects that exist in different regions, the adjacent weight spatial weight matrix is selected, and the dynamic spatial SAR, SEM, and SDM models are also introduced for spatial convergence analysis (72). The constructed model is as follows Equations (9–11):


(9) $$\begin{array}{l} SLM:\Delta lnY_{i,t+1}=\alpha +\beta \ln Y_{i,t}+\gamma X_{i,t}+\rho \sum_{j=1}^{n}w_{i,j}\Delta lnY_{i,t+1}+\\ \;c_{i}+\mu_{t}+\varepsilon_{i,t} \end{array}$$

(10) $$SEM:\Delta \text{ln}Y_{i,t+1}=\alpha +\beta \ln Y_{i,t}+\gamma X_{i,t}+c_{i}+\mu_{t}+\epsilon_{i,t}$$

(11) $$\begin{array}{l} SDM:\Delta InY_{i,t+1}=\alpha +\beta {InY}_{i,t}+\gamma X_{i,t}+\psi \sum_{j=1}^{n}w_{ij}\ln Y_{j,t}+\\ \;\rho \sum_{j=1}^{n}w_{ij}\Delta InY_{i,t+1}+\phi \sum_{j=1}^{n}w_{i,j}X_{j,t}+\\ \;c_{i}+\mu_{t}+\varepsilon_{i,t} \end{array}$$

(12) $$v=-\frac{\ln\left(1+\beta \right)}{T}$$

Where $\ln Y_{i,t}$ is the observation of the region in period *t*, $\varepsilon_{i,t}$ represents an independent and identically distributed residual term, and $\epsilon_{i,t}$ is the error term with spatial autocorrelation, and β represents the strength of the convergence process, α is a constant term. ρ is the spatial autoregression coefficien*t*, γ is spatial autocorrelation coefficient of error item, and ψ is the spatial lag coefficient of. Additionally, $w_{ij}$ is the element in spatial weight matrix, $\sum_{j=1}^{n}w_{ij}\Delta InY_{i,t+1}$ denotes the spatial interaction of $\Delta \text{In}Y_{i,t+1}$, and $c_{i}$ $\mu_{t}$ represent province and time-fixed effects. γ is the coefficient of control variable. ϕ is the spatial lag coefficient of control variable (75, 76). $X_{i,t}$ is the control variable, mainly financial self-sufficiency rate (SELF) (77, 78), urbanization rate (URB) (79, 80), openness to the outside world (OPEN) (81, 82), transportation accessibility (TRAF) (83, 84), and the level of science, technology and innovation (THNC) (85, 86), The definitions and data sources for these variables are shown in Table 4. If β<0, it means that there is β convergence. v is the rate of convergence. V is calculated from Equation (12).

**Table 4:** Definition and descriptive statistics of control variables.

Variable	Variable definition	Observes	Maximum value	Minimum value	Standard deviation	Mean
SELF	Local budgetary revenue/expenditure (%)	390	0.9314	0.1483	0.1904	0.692
URB	Proportion of urban population (%)	390	0.8958	0.2988	0.1277	0.686
OPEN	Foreign investment as a share of GDP (%)	390	0.12099	0.00006	0.0192	4.565
TRAF	Total railroad mileage in the region(10,000 km)	390	1.42	0.03	0.2278	0.301
THNC	Evaluation index of innovation capacity of Chinese regions	390	65.49	15.78	10.59	38.21

## 3 Results

### 3.1 Spatial and temporal evolution of HED

Table 5 illustrates a continuous year-by-year increase in China’s HED’s mean, rising from 0.402 in 2009 to 0.601 in 2021. This progress signifies a transition from a state of near disharmony to one of basic coordination in healthcare systems. This transformation can be primarily attributed to China’s rapid economic development over the past decade. Simultaneously, the rapid expansion of the regional economy has led to a significant upsurge in the provision of healthcare services. Notably, Guangdong Province and Jiangsu Province exhibit the highest levels of coordination, while Qinghai Province and Ningxia Province exhibit the lowest degrees of coordination.

**Table 5:** Value of HED in China from 2009 to 2021.

	2009	2010	2011	2012	2013	2014	2015	2016	2017	2018	2019	2020	2021
Anhui	0.37	0.40	0.42	0.45	0.48	0.50	0.52	0.54	0.56	0.58	0.61	0.62	0.65
Beijing	0.52	0.54	0.55	0.56	0.57	0.59	0.61	0.62	0.63	0.64	0.66	0.63	0.67
Fujian	0.42	0.44	0.46	0.49	0.51	0.53	0.54	0.55	0.57	0.59	0.60	0.60	0.63
Gansu	0.31	0.33	0.35	0.37	0.39	0.40	0.42	0.43	0.43	0.45	0.46	0.46	0.48
Guangdong	0.58	0.61	0.64	0.67	0.69	0.72	0.74	0.76	0.79	0.81	0.83	0.80	0.85
Guangxi	0.36	0.38	0.40	0.43	0.45	0.46	0.47	0.49	0.51	0.52	0.54	0.54	0.57
Guizhou	0.29	0.32	0.33	0.37	0.39	0.41	0.43	0.46	0.48	0.50	0.52	0.52	0.55
Hainan	0.29	0.30	0.31	0.33	0.35	0.36	0.37	0.38	0.39	0.40	0.42	0.42	0.44
Hebei	0.44	0.47	0.49	0.52	0.54	0.56	0.57	0.59	0.61	0.59	0.61	0.60	0.63
Henan	0.45	0.48	0.51	0.54	0.57	0.60	0.62	0.64	0.67	0.69	0.72	0.71	0.75
Heilongjiang	0.35	0.37	0.39	0.41	0.43	0.44	0.44	0.45	0.46	0.47	0.48	0.47	0.49
Hubei	0.41	0.43	0.47	0.50	0.53	0.56	0.59	0.60	0.62	0.64	0.66	0.61	0.67
Hunan	0.41	0.43	0.46	0.49	0.51	0.54	0.56	0.58	0.60	0.61	0.64	0.64	0.68
Jilin	0.36	0.38	0.38	0.42	0.43	0.45	0.45	0.46	0.47	0.48	0.49	0.49	0.51
JIANGSU	0.53	0.56	0.60	0.63	0.66	0.69	0.71	0.73	0.75	0.77	0.79	0.78	0.82
Jiangxi	0.36	0.38	0.40	0.43	0.45	0.47	0.48	0.50	0.52	0.53	0.55	0.56	0.59
Liaoning	0.44	0.46	0.49	0.51	0.53	0.54	0.52	0.52	0.53	0.54	0.55	0.54	0.56
Inner Mongolia	0.38	0.39	0.41	0.43	0.45	0.46	0.47	0.48	0.49	0.49	0.50	0.50	0.52
Ningxia	0.31	0.31	0.33	0.35	0.36	0.37	0.38	0.40	0.41	0.42	0.42	0.42	0.43
Qinghai	0.32	0.33	0.34	0.35	0.36	0.38	0.39	0.39	0.40	0.41	0.42	0.43	0.43
Shandong	0.52	0.56	0.59	0.63	0.66	0.68	0.69	0.71	0.73	0.75	0.76	0.75	0.80
Shanxi	0.39	0.40	0.41	0.43	0.45	0.46	0.47	0.48	0.48	0.50	0.51	0.51	0.53
Shanxi	0.38	0.40	0.42	0.45	0.47	0.49	0.50	0.52	0.54	0.56	0.57	0.57	0.59
Shanghai	0.52	0.54	0.56	0.56	0.57	0.59	0.61	0.62	0.64	0.65	0.66	0.65	0.68
Sichuan	0.45	0.47	0.50	0.54	0.56	0.59	0.60	0.62	0.65	0.66	0.69	0.68	0.73
Tianjin	0.41	0.43	0.45	0.46	0.48	0.50	0.50	0.51	0.51	0.51	0.52	0.51	0.53
Xinjiang	0.34	0.35	0.37	0.40	0.42	0.43	0.44	0.45	0.47	0.47	0.48	0.47	0.49
Yunnan	0.35	0.37	0.39	0.41	0.43	0.45	0.47	0.49	0.52	0.54	0.56	0.56	0.59
Zhejiang	0.51	0.54	0.57	0.60	0.62	0.64	0.66	0.68	0.71	0.73	0.75	0.74	0.78
Chongqing	0.37	0.39	0.41	0.44	0.45	0.47	0.50	0.51	0.53	0.54	0.56	0.56	0.59
Mean	0.40	0.42	0.44	0.47	0.49	0.51	0.52	0.53	0.55	0.56	0.58	0.57	0.60
Class of coordination	3	3	3	3	3	4	4	4	4	4	4	4	5

### 3.2 Spatial and temporal distribution of HED

**Figure 1:**
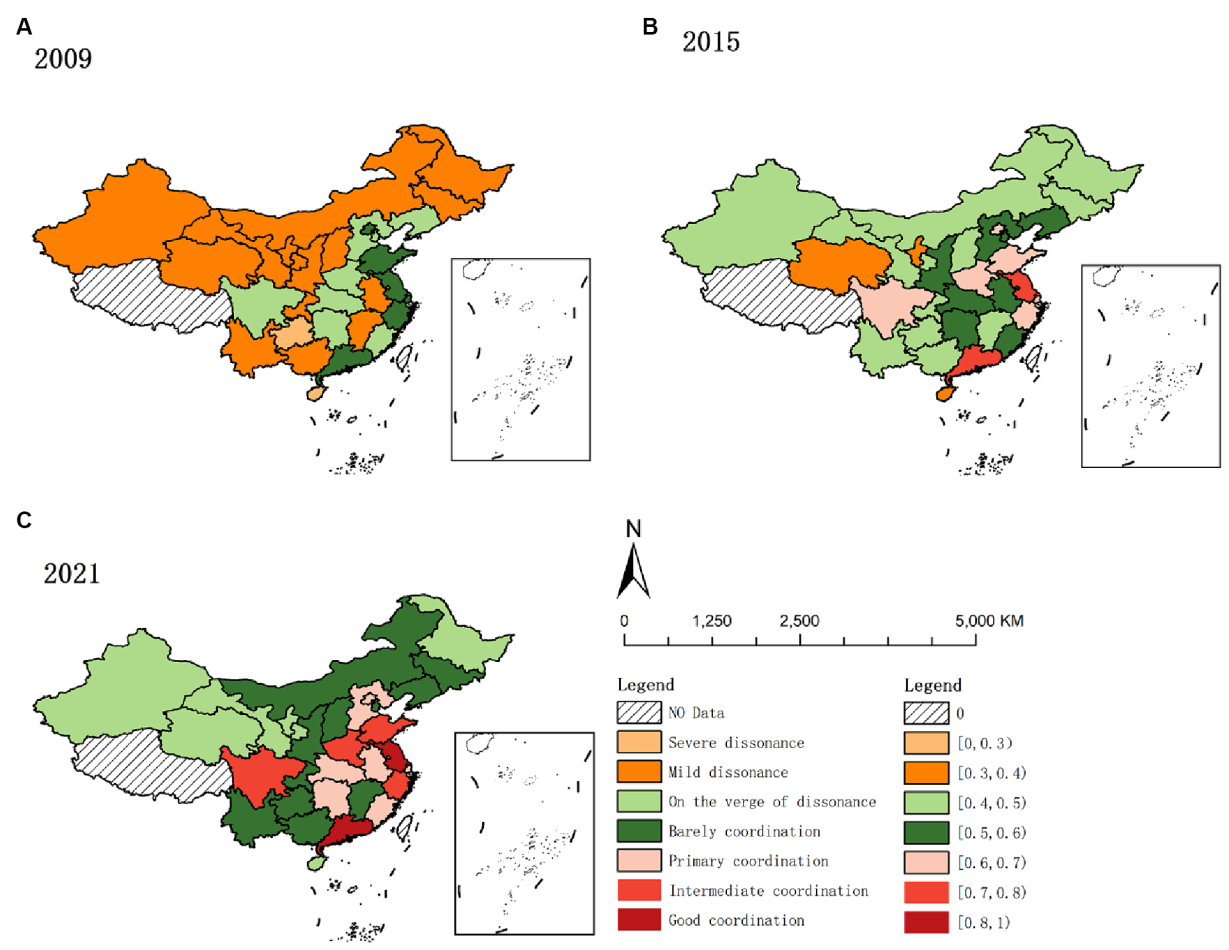
Spatial and temporal distribution of HED from 2009 to 2021. **(A)** 2009. **(B)** 2015. **(C)** 2021.

Based on the HED in 2009, 2015, and 2021, the spatial distribution and trend maps of HED in China were produced by ArcGIS software (Figure 1).

As depicted in Figure 1, high-value areas predominantly converge in the eastern region. The eastern region, compared to others, exhibits a superior economic status, a more balanced industrial structure, and a greater appeal for resources like labor and capital. These enhanced economic conditions facilitate increased investment in healthcare, improving service provision. Concurrently, improved medical services contribute to health protection and support for socio-economic development, fostering a positive interplay between economic and health outcomes. Additionally, the region’s stronger economy tends to positively influence neighboring areas, culminating in the coordinated enhancement of both medical services and the regional economy in the eastern region.

Low-value areas predominantly exist in China’s western provinces like Gansu, Qinghai, Ningxia, and Xinjiang. The western region faces dual disadvantages in geographical and economic aspects compared to other regions. Economically overshadowed by the more prosperous eastern provinces, these western provinces experience slower economic development. Concurrently, the weaker infrastructure and scarcity of medical resources contribute to slower health service delivery and economic growth. Additionally, the western provinces face harsher natural environments than the east-central region, leading to a higher demand for medical services (87). However, limited by their economic constraints, they struggle to provide adequate healthcare, resulting in a lag in coordinating medical service supply with regional economic development compared to other regions.

### 3.3 Kernel density curve estimates

**Figure 2:**
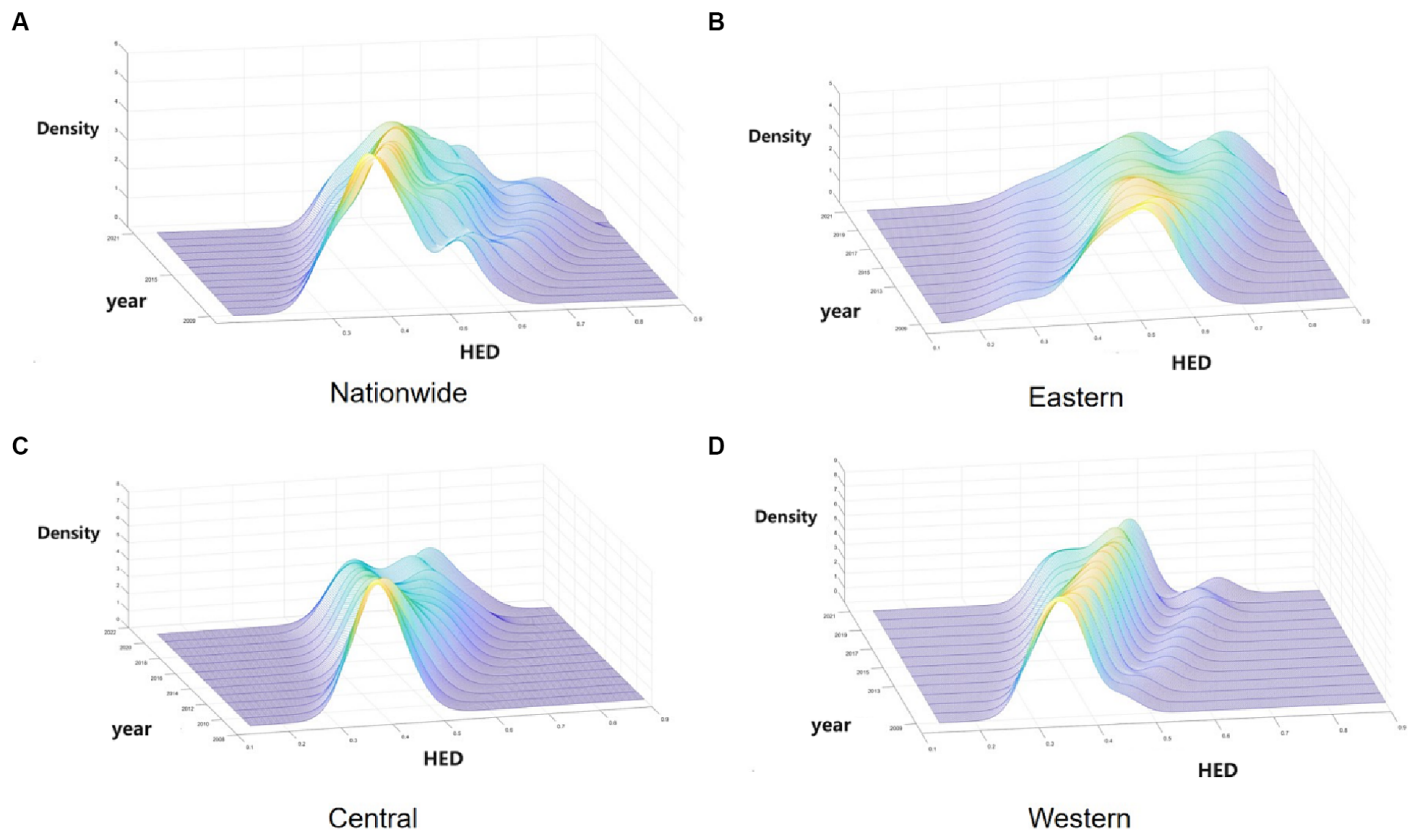
Kernel density estimation of HED in China from 2009 to 2021. **(A)** Nationwide. **(B)** Eastern. **(C)** Central. **(D)** Western.

To further investigate the dynamic evolution characteristics of HED, MATLAB software was used to perform the kernel density estimation. The results are shown in Figure 2.

As depicted in Figure 2, Figure 2A illustrates the dynamic evolution of China’s HED distribution during the survey period. Initially, the distribution’s center point progressively shifts rightward, indicating an increase in the HED. Subsequently, the distribution curve’s main peak height decreases while its width expands, suggesting a growing disparity between provinces. Thirdly, the countrywide distribution curve exhibits “right-dragging” and ongoing expansion, reflecting a widening gap between higher (e.g., Guangdong, Jiangsu) and lower (e.g., Xinjiang, Ningxia) provinces. Finally, regarding multi-peak pattern distribution, the overall trend is a “one main and two side” multi-peak configuration, indicating a notable gradient effect across China.

Secondly, Figure 2B reveals the dynamic evolution of the HED distribution in the eastern region. Initially, the east’s distribution center shifts rightward, signifying a gradual rise in HED. Subsequently, a decrease in the distribution curve’s main peak height suggests increased regional variation. Finally, from the polarization point of view, the bimodal distribution of kernel density distribution in the eastern region is gradually increasing, indicating its increasing multiploidization trend.

Thirdly, Figure 2C illustrates the dynamic evolution of the HED distribution in the central region. Initially, the curve’s center shifting rightward suggests a gradual increase in the central region’s HED. Subsequently, the continuous decline of the central distribution’s main peak reflects growing variation within the region. Finally, the curve’s evolution from “single-peaked” to a bimodal distribution in the central region indicates emerging multiploidization.

Fourthly, Figure 2D displays the dynamic evolution of the HED distribution in the western region. Initially, the western region’s coupling coordination exhibits a yearly increasing trend. Subsequently, the western region’s main peak is notably higher and narrower compared to other regions, indicating smaller absolute differences between its provinces. Finally, the western region’s shift from a single-peak to a developing double-peak curve indicates a gradual emergence of multiploidization.

Overall, China’s HED shows a rising trend, but due to the differences in the economic development rate of each province and the differences in the supply capacity of health services, the disparity in HED values within different regions is gradually becoming significant, which is manifested in the strengthening of the trend of multipolarity and the increasing of intra-regional disparities within the regions, which will also affect the balanced development of China’s overall HED.

### 3.4 Spatial pattern evolution of HED

#### 3.4.1 Global spatial pattern

Given the theoretical spatial correlation between health service supply and regional economy, spatial econometric analysis was conducted. Stata17 software calculated global Moran’s I index for 2019–2021, with results displayed in Table 6. Table 6 shows that the global Moran’s I for HED are all positive and at least 10% level of significance, indicating a significant spatial positive correlation for HED. The global Moran’s index for China’s HED increased from 0.15 to 0.252 between 2009 and 2021, indicating an evolving trend from weak to strong spatial dependence and increasingly significant spatial agglomeration in China’s HED. This may be related to China’s successive regional integration policies, such as the Yangtze River Economic Belt, the Yellow River Basin Plan and the Yangtze River Delta Integration, which have facilitated the cross-regional flow of resource factors and strengthened the economic links between regions in China. In addition, China’s high-speed railroad has been developed rapidly since 2009, narrowing the cost of transportation between regions and facilitating the flow of factors between regions (83), which has strengthened the economic and health sector links between regions.

**Table 6:** Moran’s I for HED from 2009 to 2021.

Year	Moran’s I	*Z*-value
2009	0.15 **	1.52
2010	0.17 **	1.69
2011	0.16 **	1.61
2012	0.14 *	1.45
2013	0.14 *	1.42
2014	0.15 *	1.5
2015	0.18 **	1.73
2016	0.17 **	1.71
2017	0.19 **	1.76
2018	0.19 **	1.88
2019	0.2 **	1.93
2020	0.24 ***	2.26
2021	0.25 ***	2.36

*, **, and *** indicate statistical significance at the levels of 10, 5, and 1% levels.

#### 3.4.2 Local spatial differentiation

LISA maps reflect local spatial connections between a regional unit and its neighbors, exploring spatial agglomeration characteristics of HED in each province, city, and autonomous region. Cross-sectional data from 2009, 2013, 2017, and 2021 were utilized to create LISA maps with ArcGIS software, assessing HED agglomeration and the distribution of hot and cold spots to identify potential spillover effects. The classifications include: High-High, where regions with high coordination are encircled by similar regions; Low-High, where low coordination regions are surrounded by high coordination areas; Low-Low, where low coordination regions are encompassed by others at the same level; and High-Low, where high coordination regions are surrounded by low coordination areas.

**Figure 3:**
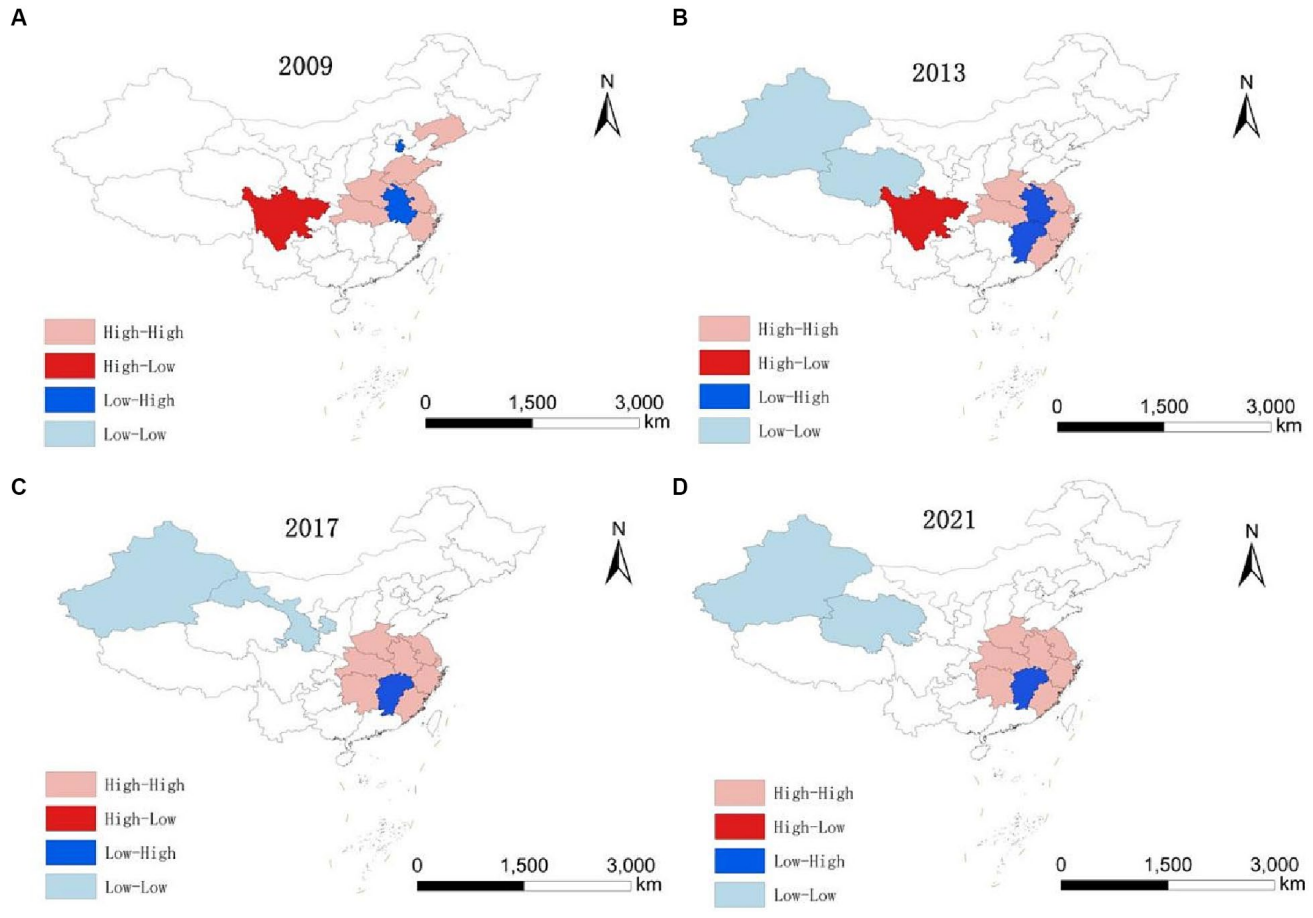
LISA evolution of HED from 2009 to 2021. **(A)** 2009. **(B)** 2013. **(C)** 2017. **(D)** 2021.

1. High-High: Figure 3 reveals two key trends in the HH region from 2009 to 2021: an expanding scope and a southward shift. In 2009, Liaoning and Shandong were HH-type, but by 2013, they lost significance, while Anhui, Hunan, and Fujian emerged as HH, likely influenced by the spatial spillover from adjacent dominant provinces, leading to increased HED in neighboring regions. This increase in HED can be attributed to the spatial spillover effect from neighboring dominant provinces. Overall, provinces in this category are predominantly situated in the eastern coastal region and along the middle and lower Yangtze River Economic Belt, central to China’s economic development. These provinces not only exhibit increasing coordination between health service supply and economic development but also are expected to develop interconnected and mutually beneficial relationships with adjacent provinces and regions.
2. Low-High: In 2009, this category included Tianjin, Anhui, and Jiangxi. By 2021, Anhui shifted from LH to HH, Tianjin became insignificant, and Jiangxi remained LH. This could be attributed to Jiangxi’s regional economy lagging behind neighboring provinces like Jiangsu and Zhejiang, resulting in a greater siphoning effect (88). Additionally, Jiangxi’s weak economic linkages with adjacent provinces (68) mean the siphoning effect outweighs the spillover effect, leading to MHSS and its regional economy trailing behind its neighbors. Anhui Province, on the other hand, due to its active integration into the Yangtze River Delta region, has fully embraced the transfer of industries in the YRD region and the diffusion effect of technology expansion in the field of health, realizing the simultaneous enhancement of the economy and the supply of health services.
3. Low-Low: From 2009 to 2021, the LL-type area predominantly encompassed Xinjiang, Ningxia, and Qinghai, with this pattern persisting throughout the period. These provinces, characterized by complex topography, slower economic growth, limited health service capacity, and fewer connections with eastern dominant regions, less exposed to spatial spillovers from high levels of HED, formed the collapse zone in China’s HED during the study period (89). In addition, this to a certain extent reflects the gap between the HED between China’s regions, the external effect of the cross-regional is not strong, the region has a certain degree of spillover effect, with a higher HED of the region can be driven to a certain extent by the development of neighboring regions, for this reason for the northwestern region, should be considered to create a high HED of the province, driven by the northwestern region of the synergistic development.
4. High-Low: From 2009 to 2013, Sichuan Province was the sole region classified as HL. Provinces in this category benefit from coordinated development in healthcare service capacity and regional economy, relative to their neighbors, and exert a driving influence. Sichuan exited the H-L category in 2017, primarily due to increased coupling and coordination in surrounding provinces and cities, and its move away from the HL.

### 3.5 Convergence characteristics of HED

#### 3.5.1 Absolute β convergence

Given the potential for varied spatial effect patterns in coupling coordination across regions, the LM test is initially applied to ascertain the presence of spatial autocorrelation in the absolute β-convergence of HED nationally and in the three regions, followed by the Hausman test, the LR test, and the Wald test to identify the most suitable spatial modeling approach.

Table 7 presents the absolute β-convergence test outcomes for HED across the nation and its three major regions. Absolute β-convergence is significant both nationally and in all individual regions. Each region’s β is negative, meeting the 1% significance threshold, suggesting a nationwide and regional trend of absolute β convergence in HED. This implies that over time, regions initially lagging in HED will grow faster than their advanced counterparts, eventually leading to uniform HED. Additionally, convergence speeds vary across regions. Nationally, the convergence rate stands at 0.015%, with a convergence period of 448.5816. Regionally, the eastern, central, and western regions exhibit convergence rates of 1.16, 1.88, and 2.09%, respectively. The western region demonstrates a markedly higher convergence rate and shorter period compared to others, whereas the eastern region lags behind the central and western regions in both aspects. This aligns with earlier analyses indicating that the eastern region’s mixed high and low coupling, alongside significant internal disparities, leads to a multipolar trend and slower convergence rate.

**Table 7:** Absolute B convergence characteristics of HED in China.

Region	National	Eastern	Central	Western
Model type	SDM	SDM	SDM	SDM
β	−0.2139***	−0.2014***	−0.2025***	−0.2226***
wxβ	0.1779***	0.1425***	0.1515***	0.1875***
ρ	0.6504***	0.50357***	0.4351***	0.6217***
*R* ^2^	0.3221	0.3235	0.2803	0.39
Log-likelihood	1041.9944	370.521	257.7257	403.7173
LM spatial error	248.486***	45.26***	30.006***	77.001***
Robust LM spatial error	1.258***	2.216***	1.5 1	0.084**
LM spatial lag	259.244***	51.24***	32.947***	81.955***
Robust LM spatial lag	12.016***	8.196**	4.511*	5.038
Hausman test	48.41***	10.47**	18.49***	81.955***
LR test spatial error	18.75***	3.27*	3.82*	12.07***
LR test spatial lag	20.2***	9.53**	6.68***	23.68***
Wald test spatial error	16.23	10.13**	7.98***	0.43
Wald test spatial lag	0.4381	4.67*	2.18	0.14
v	0.0015452	0.0116512	0.0188538	0.0209875
Time	448.5816	59.49149	36.7644	33.0267
Region fixed effect	Yes	Yes	Yes	Yes
time fixed effect	No	No	No	No
*N*	360	132	96	132

*, **, and *** indicate statistical significance at the levels of 10, 5, and 1% levels.

#### 3.5.2 Conditional β convergence

To assess conditional β-convergence in China’s HED, the model incorporates control variables, with results detailed in Table 8. The selection of spatial econometric models for various regions follows the same process as in the absolute β-convergence analysis and is not reiterated here. The results indicate that conditional β-convergence is present in both the national and the three majors regional HEDs. Specifically, the national and regional convergence coefficients β are significantly negative at the 1% confidence level. This suggests that, despite considering external factors like financial self-sufficiency, urbanization, openness, accessibility, and scientific innovation, the HED in these areas continues to converge. Furthermore, the conditional convergence of HED both nationally and regionally exceeds the absolute convergence rate, affirming the scientific validity of the chosen control variables. Lastly, spatial spillover effects are observed nationwide and across all three regions, where enhancements in regional HED positively influence neighboring areas.

**Table 8:** Condition B convergence characteristics of HED in China.

Region	National	Eastern	Central	Western
Model type	SDM	SDM	SEM	SDM
β	−0.3170***	−0.0708**	−0.3664***	−0.4314***
ρ(λ)	0.1201*	0.4177***	0.3885***	0.5052***
SELF	0.0868***	0.1309***	0.2324***	0.1696***
URB	0.1878***	−0.1071***	0.9346***	0.2053
OPEN	0.1670***	−0.0427	0.2709	−0.0228
TRAF	−0.0269*	0.0438**	−0.0745	−0.0363*
THNC	0.0004	0.0005**	0.0005	−0.0004
cons		−0.1588**		
Wxβ	0.2055***	−0.0176		0.2405***
Wx SELF	−0.0663	−0.0143		0.0202
Wx URB	−0.0183	0.0992**		0.3271*
Wx OPEN	−0.2702	−0.2681*		−2.401***
Wx TRAF	−0.0834**	0.0328		−0.1653***
Wx THNC	0.00078*	0.00039		0.0003
*R* ^2^	0.4189	0.5086	0.5407	0.6543
Log-likelihood	1130.4559	376.0898		425.2075
LM spatial error	188.913***	38.269***		50.191***
Robust LM spatial error	20.277***	6.62**		3.636**
LM spatial lag	170.042***	31.915***		48.065***
Robust LM spatial lag	1.406	0.266		1.51
Hausman test	104.14***	9.28		81.61***
LR test spatial lag	39.6 ***	14.46		52.77***
LR test spatial error	36.25***	7.9		45.06***
Wald test spatial lag	15.83***	10.98*		48.11***
Wald test spatial error	14.66**	9.44*		48.48***
v	0.0317703	0.0320431	0.0380344	0.0460114
Time	21.81748	21.63169	20.38244	15.06468
region fixed effect	Yes	No	Yes	Yes
time fixed effect	Yes	No	No	No
*N*	360	132	96	132

*, **, and *** indicate statistical significance at the levels of 10, 5, and 1% levels.

In addition, it is important to note that there are significant differences in the factors influencing HED in the country and the three major regions. Conditional β convergence analysis after adding control variables, the financial self-sufficiency rate on the national, eastern, central and western regions HED has a positive effect, and all through the 1% significance level test, which indicates that improving SELF can effectively reduce the regional differences in HED, specifically SELF improvement means that the region’s own economic hematopoietic ability to improve, and is conducive to the promotion of economic growth (90), and at the same time Fiscal revenue is the power source of medical and health service supply, and the improvement of fiscal revenue can directly promote the improvement of medical and health service supply level (91), thus forming the coordinated improvement of health service supply and regional economy; the urbanization rate has a positive impact on HED in the national and central regions, while it has a negative impact on the eastern region, and both passed the 1% significance level test, and it has a positive but not significant impact on the western region. Impact on the western region but not significant. On the one hand, it may be related to the urbanization process of each region, the urbanization rate in the eastern region is significantly higher than the national and other regions, and the continued promotion of urbanization will have a diminishing marginal benefit (92, 93), and on the other hand, the regression coefficient of urbanization is not significant in the western region, which may be due to the limited financial resources of the government of the western region, and the investment in health care in the process of urbanization is relatively small, and it fails to promote the promotion of HED effectively. Promote the improvement of HED. The openness to the outside world has a positive effect on HED at the national level, while the effect on other regions is not obvious, which may be due to the fact that the role of foreign direct investment in economic growth is still significant at the national level, but for the regional level, the promotion effect of foreign direct investment decreases with the increase in the level of economic development of the region (94); the degree of accessibility to transportation has a negative effect on the whole country and the western region, while it has a positive effect on the eastern region. Transportation accessibility has a negative promotion effect on the national and the western regions, while it has a positive effect on the eastern regions, and is not significant for the central region, which may be due to the improvement of transportation conditions, the siphoning effect of the advantaged region on the disadvantaged region is strengthened, which results in the tendency of the disadvantaged region to converge to the advantaged region has been weakened; scientific and technological innovation significantly benefits only the eastern region. This is likely due to the concentration of capital, talent, top universities, and innovation-focused companies in this region, boosting both its economy and health system (95).

## 4 Conclusions and suggestions

### 4.1 Conclusion

Current research on health service provision and regional economy primarily examines their interrelated systemic association. Secondly, Studies on spatial correlation usually use Moran’s index, but there are fewer studies on the dynamic evolution mechanism and spatial convergence of system coordination. Consequently, this paper develops a coupled coordination model linking health service supply with the regional economy. This model utilizes nonparametric kernel density and spatial econometric analysis methods to investigate the dynamic evolution and spatial convergence of this coordination. The study leads to the following conclusions:

1. In terms of the process of temporal and spatial changes in the degree of coupling coordination, firstly, the degree of coupling coordination between China’s medical and health service supply and the level of economic development has shown a good and steady upward trend during the study period. The main grade types of the coupling coordination level of each province in China have experienced a transition from being on the verge of being uncoordinated to being basically coordinated, which implies that the degree of coupling coordination between the systems is developing in a good direction. Second, there are obvious regional differences in the degree of coupling and coordination between China’s health and economic systems, showing a gradient distribution pattern of “high in the east and low in the west.” In terms of regional distribution, the eastern region has the best degree of coupling, followed by the central region, and the western region has the worst.
2. In terms of dynamic evolution, the kernel density estimation shows, first, that the centroids of the national and the three overall curves gradually shifted to the right during the study period, indicating that the degree of coupling is increasing in both the national and the three main regions; second, that the peaks are decreasing and the bandwidths are increasing, indicating an increase in the degree of variation within regions; and third, that multipolarity is occurring in all regions, suggesting that the degree of coordination of coupling within the regions is showing a gradient of differentiation.
3. In terms of spatial effects, firstly, the value of the Moran’s I index of HED in China is greater than zero in all years during the study period, indicating that the two systems have obvious spatial spillover effects on the coupling. Second, the LISA analysis shows that the distribution is characterized by “H-H” and “L-L” clustering, with H-H clustering mainly in the middle and lower reaches of the Yangtze River and L-L clustering mainly in the inland provinces of northwest China, such as Qinghai and Xinjiang. In addition, during the study period, China’s high-coupling-coordination regions showed a significant southward shift, while the low-coupling regions showed a northwestward shift.
4. From the perspective of absolute β-convergence, firstly, there is a significant β-convergence characteristic for the whole country and the regions during the study period without considering the influence of the external environment. From the perspective of absolute β-convergence, the degree of coupling coordination in the whole country and the three major regions showed β-convergence characteristics after considering economic and social factors. In addition, factors such as financial self-sufficiency rate, urbanization rate, degree of opening up to the outside world, degree of transportation convenience, and level of scientific and technological innovation have different performances in terms of their influence on the convergence of the whole country and the three major regions, and only the financial autonomy rate has a positive influence in all ranges.

### 4.2 Suggestions

Health service supply and regional economic development have a relationship of mutual influence and constraints, and the status of coupled and coordinated development is an important criterion for measuring the level of sustainable development of the two. Based on the discussion and analysis of HED in the east, middle and west of China, the following recommendations are given to promote the long-term coordinated development of the level of health services and regional economy.

1. Transform the economic development model to support new industries and promote sustainable regional economic growth. The regional economy forms the material foundation for health service provision levels. Over the past decade, China’s local economies have relied heavily on fixed asset investment, with many local governments depending largely on land transfer fees as their primary income source. However, with China’s real estate market in recession, this land finance model is increasingly unsustainable, significantly impacting local economies (96). Consequently, the Chinese government should offer policy support nationwide, encouraging gradual industrial restructuring, enhanced science and technology investment, and fostering local economic transformation and development. Local governments can further aid this transformation by supporting new industries with tax incentives, financial subsidies, and similar policies, thus spurring economic growth and establishing a robust economic base for health service supply. Additionally, local governments should manage their expenditures, curtail fixed asset investment, and enhance their financial self-sufficiency to mitigate regional disparities in HED.
2. Enhance the financial security mechanism and boost financial support for health service supply in underprivileged areas. A spatial imbalance exists between China’s fiscal revenues and health resource allocation, characterized by higher levels in the east and lower in the west. Consequently, the Chinese government should holistically evaluate each region’s economy, health service supply levels, and residents’ healthcare demands to appropriately adjust the financial distribution mechanism, fortifying financial subsidies for medical and healthcare in less developed areas (92), thereby gradually reducing regional disparities and enhancing local health service levels.
3. Bolster inter-regional exchange and collaboration to establish a synergistic development mechanism. Significant spatial β-convergence is evident in China’s HMED, along with notable spatial performance effects across all regions. Consequently, the development of economic and healthcare policies should fully consider the spatial interconnections of regions, guiding various forms of exchanges and cooperation between the economically stronger eastern provinces and the central and western regions, thereby creating an effective inter-regional transmission mechanism (97), and promoting coordinated inter-regional development through resource exchange and sharing. Additionally, such exchanges and cooperation can enable the less developed central and western regions to learn from the eastern region’s management and development practices, further improving economic and health service efficiencies in these areas.

## 5 Limitations

Owing to data collection constraints, this study analyzed only provincial-level macro data in China, without examining the relationship between health service provision and regional economic coupling and coordination at the municipal (county) level. Future research will incorporate municipal (county-level) data. This approach will allow for a more detailed understanding of the variations in coupled coordination across different levels in China.

## Data availability statement

The original contributions presented in the study are included in the article/Supplementary material, further inquiries can be directed to the corresponding author.

## Author contributions

JD: Writing – review & editing, Writing – original draft. QS: Writing – original draft, Data curation. HL: Writing – review & editing, Conceptualization. ZJ: Writing – review & editing, Conceptualization, Data curation. CG: Writing – review & editing, Data curation. DL: Writing – review & editing, Funding acquisition.
